# Protein–DNA Interactions: The Story so Far and a New Method for Prediction

**DOI:** 10.1002/cfg.303

**Published:** 2003-07

**Authors:** Susan Jones, Janet M. Thornton

**Affiliations:** EMBL –European Bioinformatics Institute Wellcome Trust Genome Campus Hinxton, Cambridge CB10 1SD UK

## Abstract

This review describes methods for the prediction of DNA binding function, and specifically summarizes a new method using 3D structural templates. The new method features the HTH motif that is found in approximately one-third of DNAbinding protein families. A library of 3D structural templates of HTH motifs was derived from proteins in the PDB. Templates were scanned against complete protein structures and the optimal superposition of a template on a structure calculated. Significance thresholds in terms of a minimum root mean squared deviation (rmsd) of an optimal superposition, and a minimum motif accessible surface area (ASA), have been calculated. In this way, it is possible to scan the template library against proteins of unknown function to make predictions about DNA-binding functionality.


**Comparative and Functional Genomics**
*Comp Funct Genom* 2003; **4**: 428–431.
Published online 18 July 2003 in Wiley InterScience (www.interscience.wiley.com). **DOI:** 10.1002/cfg.303

**Conference Review**

### Protein–DNA interactions: the story so far and a new method for prediction

Susan Jones* and Janet M. Thornton
*EMBL–European Bioinformatics Institute, Wellcome Trust Genome Campus, Hinxton, Cambridge CB10 1SD, UK*

**Correspondence to:*
*Susan Jones, EMBL–European Bioinformatics Institute, Wellcome Trust Genome Campus, Hinxton, Cambridge CB10 1SD, UK.*
*E-mail: suej@ebi.ac.uk*

Received: 7 May 2003
Revised: 30 May 2003
Accepted: 30 May 2003

### Abstract

**This review describes methods for the prediction of DNA binding function, and specifically summarizes a new method using 3D structural templates. The new method features the HTH motif that is found in approximately one-third of DNA-binding protein families. A library of 3D structural templates of HTH motifs was derived from proteins in the PDB. Templates were scanned against complete protein structures and the optimal superposition of a template on a structure calculated. Significance thresholds in terms of a minimum root mean squared deviation (rmsd) of an optimal superposition, and a minimum motif accessible surface area (ASA), have been calculated. In this way, it is possible to scan the template library against proteins of unknown function to make predictions about DNA-binding functionality. Copyright © 2003 John Wiley & Sons, Ltd.**

**Keywords:** DNA-binding; Helix-turn-helix; motif; structural template

### Background — the story so far

The 3D structures of over 660 proteins bound to DNA molecules have been determined [Nucleic Acid Database (NDB): version 23 April 2003 [4]]. These proteins have diverse structural folds, and achieve binding and recognition of specific sites on nucleic acids in many different ways. Protein–DNA interactions are critical for the flow of biological information from genes to proteins, and have consequently been the focus of considerable research. Much of this has involved the description of specific complexes (for review of recently solved structures, see [1]) and of families of proteins sharing the same DNA binding motif (e.g. [6,2,19]).

With the large number of protein–DNA complexes deposited in the Protein Data Bank (PDB) [5] and curated in the NDB [4], it has been possible to analyse large sets of non-homologous complexes and derive general characteristics of DNA binding sites on proteins [10,14,12]. These sites comprise discontinuous sequence segments forming one or more hydrophilic surfaces capable of direct and water-mediated hydrogen bonds. The extent of the binding site varies widely [618–2833 Å$^2$ accessible surface area (ASA) per monomer] and most sites are rich in lysine and arginine residues [10,14].

Proteins binding to DNA commonly force structural deformation upon both parts of the complex. The deformation of the DNA, usually described as DNA bending, has been extensively studied (e.g. [15]). Forced bending commonly occurs through specific kinks of the double helix, generally at pyrimidine–purine base steps [7]. In comparing bound and unbound DNA molecules the deformations in bound DNA were observed to be more extreme than those of unbound DNA [10]. The conformational change in the protein can also be substantial with disorder-to-order transitions, domain movements and quaternary changes all documented [14].

With the recent development of structural genomics projects in which protein structures are solved that have very low sequence identity (and potentially little or no fold similarity) to any currently in the PDB [5], the number of DNA binding proteins in the PDB can only be set to

Copyright © 2003 John Wiley & Sons, Ltd.

increase. This will provide further structures for analysis, but more importantly gives rise to a need for methods that predict the potential DNA-binding function of a new structure that has little or no structural similarity to any currently known.

Methods for the prediction of protein–DNA interactions fall into two categories, the prediction of the DNA sequence bound given a protein binding site, and the prediction of a DNA binding site on the protein given the unbound structure. The first category has been addressed using pairwise potentials that estimate the likelihood of a amino acid making favourable contacts with a DNA base [13,11]. The second category of prediction is more pertinent to the problems faced by structural genomics projects that require fast and reliable methods for the prediction of protein function, and has only recently been addressed.

The paper by Stawiski *et al.* [18] presents an automated method for the prediction of DNA-binding proteins, using a combination of features derived for electrostatic patches on the protein surface. The method uses a neural network to discriminate between DNA-binding and non-DNA binding positive electrostatic patches, using parameters such as surface area, hydrogen bonding potential, amino acid composition, surface concavity and sequence conservation. The method predicts DNA-binding proteins with high accuracy, and is capable of predicting those with novel binding motifs, and those solved in an unbound state. This is the first automated prediction method that has been successfully applied to a large data set.

In contrast to the complex method of Stawiski *et al.* [18], a relatively simple and fast method is now presented that is based on the assessment of the superposition of 3D structural templates of DNA-binding motifs on complete protein structures [9]. The method uses the HTH motif as a prototype template, but it is envisaged that the method is applicable to other DNA-binding motifs. The simplicity of the method has allowed it to be set up as a web server (**http://www.ebi.ac.uk/thornton-srv/databases/DNA-motifs**), which allows users to upload published and proprietary protein structures for the prediction of DNA-binding function.

### A new method for prediction using structural templates

The start point for the new method was a list of 86 non-identical proteins from the PDB known to contain at least one HTH motif. The list was derived from a combination of searches with Hidden Markov Models from multiple sequence alignments in Pfam [3] and SMART [17] and initial structure database searches [9]. These proteins were clustered into seven fold families (H-level) using CATH [16], and the structure with the highest resolution was taken as a representative.

For each representative an HTH motif template was created. A template is a set of $C_\alpha$ backbone coordinates of an HTH motif, sequentially continuous in terms of residue number, and comprising all the residues from two residues preceding H1 to two residues succeeding H2. The templates were scanned against whole protein structures using an algorithm that computed a gapless optimal superposition. The match of a template on a complete protein was taken as the minimum rmsd obtained from all possible superpositions.

The seven templates were scanned against (a) the 86 non-identical HTH containing structures (termed *HTH × TRUE*) and (b) the 8264 non-identical structures in the CATH database that excluded the known HTH proteins (termed *HTH × FALSE*). In each case the rmsd recorded for each structure was the minimum value calculated from any of the templates (excluding self-matches). The distribution of rmsd values is shown as a histogram in Figure 1. Using this data, a threshold value (below which a protein was predicted to contain a DNA-binding HTH motif) was selected at 1.6 Å. At this threshold there are 0.7% (61/8264) false positives, i.e. proteins predicted to include a DNA-binding HTH motif but not known to do so. This threshold also gave 11.6% (10/86) false negatives, i.e. proteins known to include a DNA-binding HTH motif but predicted as not containing one, and 88.4% (76/86) true hits.

The number of false positives was reduced by analysing the accessible surface area (ASA) of the residues comprising the HTH templates using NACCESS [8]. The absolute ASA for the residues in the 86 non-identical HTH templates ranged from 992 $Å^2$ to 2740 $Å^2$. A minimum ASA value for a DNA binding HTH motif was set at 990 $Å^2$. Using this value, the number of false positive proteins

Copyright © 2003 John Wiley & Sons, Ltd. 

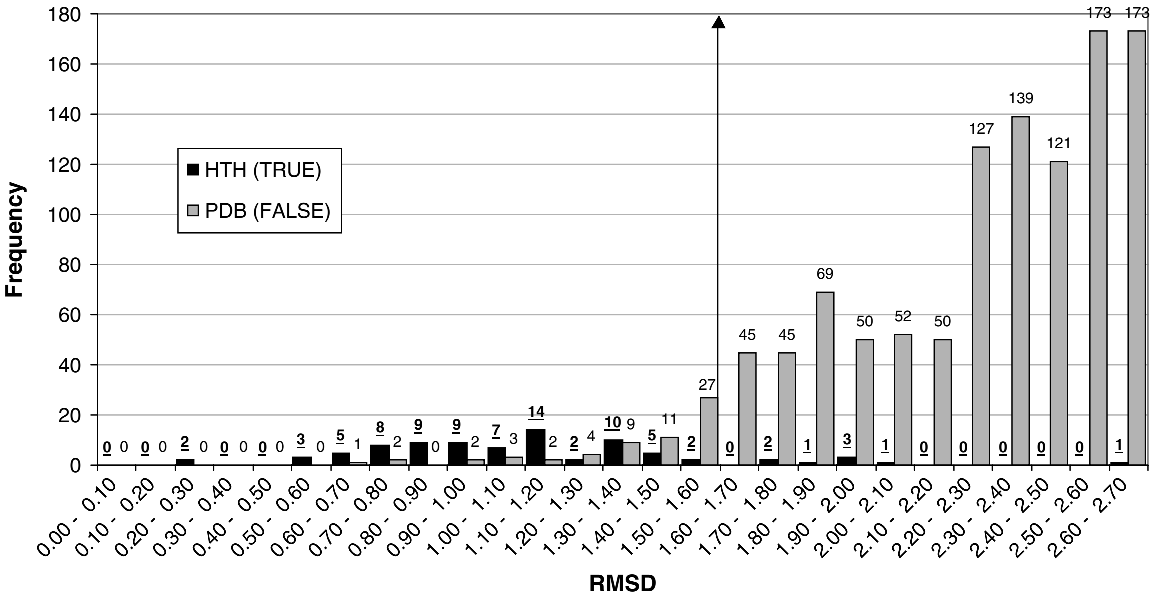

**Figure 1.** Frequency histogram showing the distribution of rmsd values resulting from the scan of seven HTH templates against 86 HTH proteins (*HTH* × *TRUE*) (shown in black) and 8264 PDB proteins (excluding known HTH proteins) (*HTH* × *FALSE*) (shown in grey). A threshold value is indicated at 1.6 Å, below which a protein is predicted to contain a DNA-binding HTH motif (note: the maximum rmsd shown is 2.7 Å but the distribution for *HTH* × *FALSE* extended to 6.1 Å)

was reduced to 0.5% (38/8264). Of the remaining 38 structures classified as false positive matches, three structures were predicted to included new HTH motifs, polymerase I (1taq), histone acetyltransferase (1fy7) and methyltransferase (1mgt).

To demonstrate the potential of the method, the template library was scanned against 30 structures from the Midwest Center for Structural Genomics (MCSG) Initiative (**http://www.mcsg.anl.gov**). One structure (target APS048) was predicted to have an HTH motif involved in DNA binding. This target (1 mkm) is the structure of *T. maritima* 0065, a member of the IcIR (isocitrate lyase regulator) transcriptional factor family [20]. It is now known that the N-terminal domain of the structure has a DNA binding function, with a HTH-motif comprising H2 and H3 with a four-residue turn between them [20]. This motif is the one matched by a template at position 21–44 of the target.

### Discussion

This new method of using 3D structural templates to make predictions about the potential DNA-binding function of proteins has been successfully used to make predictions for structural genomics targets. However, the functionality of any new prediction method will be measured by its independence from overall fold similarity. For the current method the occurrence of matches between templates derived from structures of one fold family and complete structures from a different fold family clearly demonstrates the methods independence of fold similarity (Figure 2).

Methods such as the one described here (and more fully elsewhere [9]) and that recently published by Stawiski *et al.* [18] are amongst the first to address the issue of predicting DNA-binding function. These, and other new methods, will be an integral part of a larger prediction system that will be capable of making inferences on function, from the presence of binding clefts, and the identification of enzyme active sites and small molecule binding sites.

### Acknowledgements

We would like to thank Professor Helen Berman, Irene Nobeli and Jonathan A. Barker for their contributions to the HTH motif project. S.J. was supported by a US Department of Energy Grant (DE-FG02-96ER62166).

Copyright © 2003 John Wiley & Sons, Ltd. 

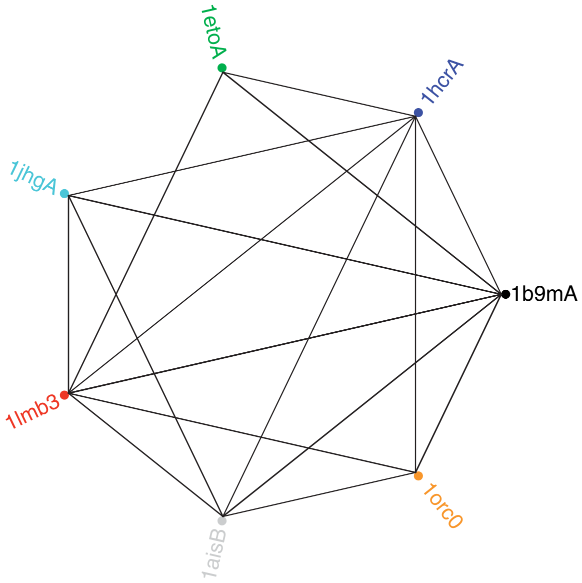

**Figure 2.** Wheel diagram depicting the identification of HTH motifs using structural templates. The seven proteins from which motifs were derived, are representatives of different fold families. A line joining two PDB codes indicates the successful match of one structure's template against the complete structure of the second protein. A successful match was taken as one where a maximal superposition gave an rmsd < 1.6 Å. The diagram effectively shows that the templates are generic, identifying structures from more than one fold family

### References

1. Aggarwal AK, Doudna JA. 2003. Protein–nucleic acid interactions: Editorial overview. *Curr Opin Struct Biol* **13**: 3–5.
2. Aravind L, Landsman D. 1998. AT-hoot motifs identified in a wide variety of DNA-binding proteins. *Nucleic Acids Res* **26**: 4413–4421.
3. Bateman A, Birney E, Cerruti L, *et al.* 2002. The Pfam protein families database. *Nucleic Acids Res* **30**: 276–280.
4. Berman HM, Olson WK, Beveridge DL, *et al.* 1992. The Nucleic Acid Database: a comprehensive relational database of three-dimensional structures of nucleic acids. *Biophys J* **63**: 751–759.
5. Berman HM, Westbrook J, Feng Z, *et al.* 2000. The Protein Data Bank. *Nucleic Acids Res* **28**: 276–280.
6. Burley SK. 1994. DNA-binding motifs from eukaryotic transcription factors. *Curr Opin Struct Biol* **4**: 3–11.
7. Dickerson RE. 1998. DNA bending: the prevalence of kinkiness and the virtues of normality. *Nucleic Acids Res* **26**: 1906–1926.
8. Hubbard SJ. 1993. NACCESS. Department of Biochemistry and Molecular Biology, University College, London.
9. Jones S, Barker JA, Nobeli I, Thornton JM. 2003. Using structural motif templates to identify proteins with DNA binding function. *Nucleic Acids Res* **31**: 2811–2823.
10. Jones S, van Heyningen P, Berman HM, Thornton JM. 1999. Protein–DNA interactions: a structural analysis. *J Mol Biol* **287**: 877–896.
11. Kono H, Sarai A. 1999. Structure-based predictions of DNA target sites by regulatory proteins. *Proteins* **35**: 114–131.
12. Luscombe NM, Laskowski RA, Thornton JM. 2001. Amino acid–base interactions: a three-dimensional analysis of protein–DNA interactions at an atomic level. *J Mol Biol* **29**: 2860–2874.
13. Mandel-Gutfreund Y, Margalit H. 1998. Quantitative parameters for amino-base interaction: implications for prediction of protein–DNA binding sites. *Nucleic Acids Res* **26**: 2306–2312.
14. Nadassy K, Wodak SJ, Janin J. 1999. Structural features of protein–nucleic acid recognition sites. *Biochemistry* **38**: 1999–2017.
15. Olson WK, Gorin AA, Lu XJ, Hock LM, Zhurkin VB. 1998. DNA sequence-dependent deformability deduced from protein–DNA crystal complexes. *Proc Natl Acad Sci USA* **95**: 11 163–11 168.
16. Orengo CA, Michie AD, Jones S, Jones DT, Swindells MB, Thornton JM. 1997. CATH — a hierarchic classification of protein domain structures. *Structure* **5**: 1093–1108.
17. Schultz J, Milpetz F, Bork P, Ponting CP. 1998. SMART, a simple modular architecture research tool: identification of signalling domains. *Proc Natl Acad Sci USA* **95**: 5857–5864.
18. Stawiski EW, Gregoret LM, Mandel-Gutfreund Y. 2003. Annotating nucleic acid-binding function based on protein structure. *J Mol Biol* **326**: 1065–1079.
19. Tateno M, Yamasak K, Amano N, *et al.* 1998. DNA recognition by beta-sheets. *Biopolymers* **44**: 335–359.
20. Zhang RG, Kim Y, Skarina T, *et al.* 2002. Crystal structure of *Thermotoga maritima* 0065, a member of the IclR transcriptional factor family. *J Biol Chem* **277**: 19 183–19 190.

Copyright © 2003 John Wiley & Sons, Ltd. 
